# Prevalence and associated factors of recurrent pregnancy loss in Nigeria according to different national and international criteria (ASRM/ESHRE vs. WHO/RCOG)

**DOI:** 10.3389/frph.2023.1049711

**Published:** 2023-02-21

**Authors:** George Uchenna Eleje, Emmanuel Onyebuchi Ugwu, Emeka Philip Igbodike, Divinefavour Echezona Malachy, Ekeuda Uchenna Nwankwo, Joseph Odirichukwu Ugboaja, Joseph Ifeanyichukwu Ikechebelu, Uchenna Ifeanyi Nwagha

**Affiliations:** ^1^ Effective Care Research Unit, Department of Obstetrics and Gynaecology, Nnamdi Azikiwe University, Nnewi, Nigeria; ^2^ Institute of Maternal and Child Health, College of Medicine, University of Nigeria Ituku-Ozalla Campus, Enugu, Nigeria; ^3^ Department of Obstetrics and Gynaecology, Nnamdi Azikiwe University Teaching Hospital Nnewi, Nnewi, Nigeria; ^4^ Department of Obstetrics and Gynaecology, College of Medicine, University of Nigeria Ituku-Ozalla, Enugu, Nigeria; ^5^ Rural Community Clinical School, School of Medicine, Deakin University, Melbourne, VIC, Australia

**Keywords:** abortions, antiphospholipid syndrome, counselling, miscarriage, recurrent

## Abstract

**Background:**

In low-and middle-income countries, no conclusive research explains the prevalence and associated factors of women with a history of recurrent pregnancy loss (RPL). Some authorities have recommended further scientific research on the effect of various definitions of RPL.

**Objective:**

To assess prevalence and associated factors of RPL among pregnant women in Nigeria according to different national and international criteria: the American Society for Reproductive Medicine/ European Society for Human Reproduction and Embryology (ASRM/ESHRE; two losses) and the World Health Organization/ Royal College of Obstetricians and Gynecologists (WHO/RCOG; three consecutive losses) criteria.

**Methods:**

This is a cross-sectional analytical study wherein, pregnant women with prior RPL were investigated. The outcome measures were prevalence and risk factors. The associations between independent variables and outcome variable were explored using bivariate and multivariable logistic regression models. The results of these analyses were reported as adjusted odds ratios (AORs) with 95% confidence intervals (95%CI). Factors associated with RPL were identified using multivariate regression models.

**Result:**

Of the 378 pregnant women interviewed, the overall prevalence of RPL in this study was found to be 15.34% (95% confidence interval = 11.65%–19.84%). The prevalence of RPL was 15.34% (58/378; 95%CI = 11.65%–19.84%) and 5.29% (20/378; 95%CI = 3.23%–8.17) according to the ASRM and the WHO criterion respectively. Regardless of diagnostic criteria, unexplained (AOR = 23.04; 95%CI: 11.46–36.32), endocrine disturbances (AOR = 9.76; 95%CI: 1.61–63.19), uterine abnormalities (AOR = 13.57; 95%CI: 3.54–50.60), and antiphospholipid syndrome (AOR = 24.59; 95%CI: 8.45–71.04) were positively and independently associated with RPL. No significant risk factors were seen when the ASRM/ ESHRE criterion vs. WHO/RCOG criterion were compared. Advanced maternal age was significantly higher in secondary than in primary type of RPL.

**Conclusion:**

The prevalence of RPL was 15.34% and 5.29% according to ASRM/ESHRE and WHO/RCOG criterion respectively, with secondary type predominating. No significant differences with regard to risk factors were seen according to diagnostic criteria studied, though advanced maternal age was significantly higher in secondary RPL. Further research is needed to confirm our findings and to better characterize the magnitude of differences.

## Introduction

The definition of recurrent pregnancy loss (RPL) varies and has been debated among international societies (1, 2). For the World Health Organisation (WHO), and the Royal College of Obstetricians and Gynecologists (RCOG), RPL refers to three consecutive pregnancy losses, including nonvisualized ones (3). However, according to the American Society for Reproductive Medicine (ASRM) and the European Society for Human Reproduction and Embryology (ESHRE), it is defined as two or more clinical pregnancy losses (documented by ultrasound or histopathologic examination), but not necessarily consecutive (2–4).

Epidemiological studies of RPL are important to gain an understanding of the disorder and its occurrence in the population. In previous studies, RPL has been reported to affect up to 2% to 5% of couples (5, 6). In a very recent Swedish study by Rasmark et al., the authors suggested that it would be interesting to compare the frequencies of three consecutive miscarriages with two (7). The prevalence of RPL can also vary widely between reports because of the differences in whether the RPL is primary or secondary. Primary RPL refers to multiple losses in a pregnant woman without viable previous babies, while secondary RPL refers to multiple losses in a woman who has already had a pregnancy beyond age of viability (8). The determination of the prevalence of RPL is helpful for planning clinical investigations and treatment protocols or for cost-benefit designs for allocating resources for reproductive care.

The established risk factors of RPL include endocrine, anatomical, infection-related, genetic, hemostasis-related and immunological factors (9). In a previous study by Youssef et al., aimed at determining whether the distribution of RPL-associated factors was different in women with two vs. three or more pregnancy losses, no associated factor was found in 71.5% of couples with RPL and these did not differ statistically between women with two vs. three or more pregnancy losses (10). The distribution of investigated causes did not differ between the two groups too (10). In one systematic review and meta-analysis by van Dijk et al., it was revealed that a difference in prevalence in uterine abnormalities and antiphospholipid syndrome, chromosomal abnormalities, inherited thrombophilia and thyroid disorders was not seen in women with two vs. three pregnancy losses (11).

In low- and middle-income countries like Nigeria, no conclusive research explains the prevalence and associated factors of women with a history of RPL in the region. Furthermore, according to the most recent RPL guideline from the ASRM and the ESHRE, it recommended that RPL could be considered after the loss of two or more pregnancies and stresses the importance of further scientific research, including epidemiological studies on the effect of various definitions of RPL (12). Therefore, the general objective of this research is to test the hypothesis that there is no significant difference in the prevalence of RPL and its associated risk factors when diagnosed according to the ASRM/ESHRE criteria vs. the WHO/RCOG criteria.

## Methods

### Study period and area

The study was carried out from December 1, 2021, to May 31, 2022, at the Nnamdi Azikiwe University Teaching Hospital (NAUTH), Nnewi, Nigeria. The hospital was selected for the survey, as it is a multidisciplinary well equipped tertiary hospital with adequate pregnant women from all over Anambra state and its environs, and also a working center for the lead author. The research hospital also had a total of 44 consultants Obstetrician-gynecologist and so were equipped for management of RPL.

### Study design and population

An institutional-based cross-sectional analytical study design was applied. The study was conducted following the Strengthening the Reporting of Observational studies in Epidemiology (STROBE) statement for reports of cross-sectional studies recommendations. All booked pregnant women attending antenatal care at NAUTH, Nnewi, Nigeria during the study period were the study population.

### Inclusion and exclusion criteria

All consenting pregnant women at a gestational age of 20 weeks and below were included. Post natal women, pregnant women with history of one or more induced or spontaneous abortions and pregnant women who are unsure of their date and without early (≤20 weeks) dating ultrasound were excluded from the study.

### Sample size

The sample size was 346, which was determined using the Cochran formula: *n* = (*z*^2^*p*q)/*e*^2^; where *p* is the prevalence of secondary RPL taken from the previous study by Ticconi et al*.* in Italy, i.e., 34.1% (13), *z* is 1.96 at 95% confidence level, *q* is (1 − *p*), and e is the error margin, i.e., 5% and 381 when we considered 10% attrition rate.

### Sampling procedure

The study participants were selected by simple random sampling using lists (sampling frame) of pregnant women from each selected unit.

### Data collection tools, procedure and quality assurance

At the time of data collection, all participants were informed about the study and its objectives and informed written consent was obtained from those participants willing to volunteer for the study. The researchers had surveyed the participants physically using the pre-structured interview questionnaire in an English format by translating it into vernacular without disturbing the actual meaning of the sentence. The translation of the questionnaire was completed by the principal investigator.

Data collection questionnaire were modified from previous similar studies. Data was collected by using pre-tested and self-administered questionnaire design in English. The tool includes three sections; socio-demographic characteristics, life style, and personal habits, and reproductive and menstrual history were included. Data collectors explained the purpose of this study to study participants and have obtained consent from participants prior to data collection. The questionnaire was pre-tested at the NAUTH, Nnewi, Nigeria, using 10 pregnant women. A one-day training was given for data collectors and supervisors on objective of the study, methods of data collection, handling of data and ways of approaching the respondents. Trained nurses measured height and weight, using the stadiometer (Model RGZ-160, China) with participants wearing no shoes. The principal investigator checked the activities of each data collector and daily checked the completeness and clarity of the questionnaires during data collection period.

### Operational definition

For the World Health Organisation (WHO) and the Royal College of Obstetricians and Gynecologists (RCOG), RPL refers to three consecutive pregnancy losses, including nonvisualized ones. However, according to the American Society for Reproductive Medicine (ASRM) and the European Society for Human Reproduction and Embryology (ESHRE), it is defined as two or more clinical pregnancy losses (documented by ultrasound or histopathologic examination), but not necessarily consecutive (2). Primary RPL refers to multiple losses in a pregnant woman with no previous viable infants, whereas secondary RPL refers to multiple losses in a woman who has already had a pregnancy beyond 28 weeks of gestation (7). Advanced maternal age was defined as an age greater than 35 years. Uterine anomalies were defined as cervical weakness, fuse intrauterine connections or uterine synechiae or Asherman's syndrome, uterine myomas, and/or endometrial polyps. Endocrine factors consist of diabetes mellitus, thyroid dysfunction, prolactin abnormalities, and/or polycystic ovary syndrome. Previous psychological pressure included maternal stress during prior pregnancies. Ectopic and molar pregnancies were excluded from the definition of recurrent pregnancy loss, whereas pregnancy loss after spontaneous conception and assisted reproductive treatment were included in the definition of RPL.

### Study variables

The dependent variable in this study was prior recurrent pregnancy loss. Independent variables included the following; sociodemographic characteristics (age of the participant, marital status, level of education, occupation of the partner, socioeconomic class, and smoking status of the participant), obstetric factors (parity, primiparity [those who have delivered once], multiparity [between 2 and 4 deliveries] and grand multiparity [greater than or equal to 5 deliveries]), as well as the body mass index (BMI). Social class stratification was determined according to Olusanya et al. (14): classes 1, 2, and 3 were considered upper class, middle class and lower social class, respectively. Tertiary education was defined as polytechnic or university education. The body mass index (BMI) was calculated by dividing the weight with the square of the height and the quotient expressed in kg/m^2^ (WHO, 2000). The BMI was then interpreted and classified as underweight (less than 18.5 kg/m^2^); normal (18.5–24.9 kg/m^2^); overweight (25–29.9 kg/m^2^) and obese (30 kg/m^2^ and above) (15). Dependent variables included history of previous pregnancy losses, medical factors (body mass index, history of any of the following: uterine anomalies, endocrine disorders, psychological pressure, antiphospholipid antibody syndrome, and unexplained factors.

### Methods of data processing and analysis

After data collection, the data was cleaned and coded before data entry. Excel Spreadsheet version 2013 was used for data entry and exported to Statistical Package for Social Sciences version 25 (IBM, Armonk, NY, USA) for analysis. Descriptive summary was used to describe the characteristics of the participants in terms of frequencies, proportion, mean and standard deviation, and the information was presented by text and tables. Socio-demographic data and severity of symptoms in women with prior RPL were compared with those of women without prior RPL. Prevalence was reported as percentage with a 95% confidence interval (95% CI). The logistic regression model of bivariate and multivariate analysis was used to identify factors associated with the outcome variable and were expressed in odds ratios (ORs). In bivariate analysis, all variables with *p*-value less than 0.05 were considered as a candidate for multivariable analysis. For the construction of logistic regression models to determine the associated factors for RPL, the dependent variable was the presence or absence of RPL. This was put against all the variables it depended upon. These significant factors were put in a model, and factors were removed one by one to produce best-fit multiple logistic models. An adjusted odds ratio (OR) with 95% confidence intervals and a *p*-value less than 0.05 was considered as statistically significant association.

### Ethical approval

This study was approved by the NAUTH Ethics Committee, Nnewi, Nigeria on March 17, 2021, with a reference number NAUTH/CS/66/VOL.14/VER.3/06/2020/081.

## Results

### Socio-demographic characteristics of the respondents

A total of 3,961 women attended the antenatal clinic during the study period. Of these, 542 were assessed for eligibility to participate in the study. One hundred twenty-four participants whose gestational age was more than 20 weeks, 11 that had at least one previous induced or spontaneous pregnancy loss and 29 participants who came for their post natal visit were excluded from the study. Therefore, 378 women were enrolled in the study and were screened for previous recurrent pregnancy loss (RPL), including 58 that had prior RPL and 320 participants without prior RPL. Of the 378 pregnant women interviewed**,** the overall prevalence of RPL in this study was found to be 15.34% (95% confidence interval = 11.65%–19.84%).

The age ranges from 18 to 42 years with a mean of 31.72 (SD ± 5.10) years. The mean age for those with prior RPL was 32.24 (SD ± 4.65) years while those without prior RPL were 31.63 (SD ± 5.18) years (*p* = 0.501). Table 1 shows the bi-variable logistic regression of the sociodemographic distribution of participants across research groups. Most 241 (63.76%) of the participants were classified into the age group of 26–35 years. Most of the participants 370 (97.88%) were married and the majority of the participants 335 (88.62%) had at least secondary level of education. Approximately 20.0% of the study participants were of upper social class.

**Table 1:** Bi-variable logistic regression of sociodemographic distribution of participants across research groups.

		Prior RPL (*n* = 58)	No prior RPL (*n* = 320)	Total	OR(95% CI)	*p*-value
Smoking status	No	57(98.5)	313 (97.8)	370 (97.9)	0.78 (0.09–6.50)	0.82
	Yes	1 (1.5)	7 (2.2)	8 (2.1)	*r*	*r*
Age range (years)	<25	6(10.4)	36(11.2)	42(11.1)	0.65 (0.21–2.16	0.49
	26–35	34(58.6)	205(64.1)	239(63.2)	0.55 (0.27–1.13)	0.10
	36–45	18(31.0)	79(24.7)	97(25.7)	*r*	*r*
	>45	0(0.0)	0(0.0)	0(0.0)		
Marital status	Married	57 (98.3)	313 (97.8)	370 (97.9)	1.53 (0.15–14.88)	0.71
	Single	1 (1.7)	7 (2.2)	8 (2.1)	*r*	*r*
Level of education	No formal education	0 (0.0)	8 (2.5)	8 (2.1)	<0.001 (<0.001–<0.001	<0.001
	Primary	10 (17.2)	25 (7.8)	35 (9.3)	4.27 (1.24–14.65)	0.021
	Secondary	24 (41.4)	181 (56.6)	205 (54.2)	0.66 (0.21–2.01)	0.46
	Tertiary	24 (41.4)	106 (33.1)	130 (34.4)	*r*	*r*
Social status	Upper class	12 (20.7)	48 (15.0)	60 (15.9)	2.38 (0.85–6.67)	0.09
	Middle class	26 (44.8)	85 (26.6)	111 (29.4)	1.00 (0.31–3.15)	1.00
	Lower class	20 (34.5)	187 (58.4)	207 (54.8)	*r*	*r*
Parity	Nulliparous	14 (24.1)	13 (4.1)	27 (7.2)	7.38 (2.19–24.88)	<0.001
	1–4 births	36 (62.1)	276 (86.2)	312 (82.5)	0.57 (0.23–1.41)	0.23
	>4 births	8 (13.8)	31 (9.7)	39 (10.3)	*r*	*r*
BMI	Underweight	0 (0.0)	0 (0.0)	0 (0.0)	–	–
	Normal	19 (32.8)	104 (32.5)	123 (32.5)	0.48 (0.22–1.06)	0.07
	Overweight	16 (27.6)	129 (40.3)	145 (38.4)	0.57 (0.26–1.21)	0.42
	Obese	23 (39.6)	87 (27.2)	110 (29.1)	*r*	*r*

Note that reference category = RPL, recurrent pregnancy loss; BMI, body mass index.

### Prevalence of recurrent pregnancy loss according to different international criteria and types

The overall prevalence of RPL in this study was found to be 15.34% (95% confidence interval = 11.65%–19.84%). This study identified that according to the ASRM criterion, 15.34% (95% confidence interval = 11.65%–19.84%) of participants had at least two previous RPL, while according to the WHO criterion, 5.29% (95% confidence interval = 3.23%–8.17) of the participants had at least three previous RPL. The comparison between such results revealed a significant difference of 10.05% (95% confidence interval = 7.11%–13.80%).

However, among those who had RPL, 15.52% (95% confidence interval = 7.10%–29.46%) had primary RPL while 84.48% (95% confidence interval = 62.50%–100.00%) had secondary RPL. Comparison between such results revealed a significant difference of 68.97% (95% confidence interval = 49.27%–93.91%).

### Factors associated with recurrent pregnancy loss

A history of unexplained RPL, endocrine disorder, uterine anomalies, psychological pressure, and antiphospholipid syndrome were significantly associated with RPL in bivariate logistic regression analysis (Table 2). All these variables with a *p*-value of <0.05 in the bivariate analysis were entered to multivariable logistic regression analysis.

**Table 2:** Bi-variable and multivariable logistic regression of risk factors for RPL in the whole participants.

	Prior RPL (*N* = 58)	No prior RPL (*N* = 320)	Total (*N* = 378)	OR (95%CI)	*p*-value	aOR^a^ (95%CI)^f^	*p*-value^f^
Unexplained	34(58.6)	0 (0.0)	34 (9.0)	90.63 (53.70–100.00)	<0.001	23.04 (11.46–36.32)	0.001
Advanced maternal age	18 (31.0)	79 (24.7)	97 (25.7)	1.25 (0.81–1.93)	0.31	–	–
Previous history of endocrine disorder	6 (10.3)	2 (0.6)	8 (2.1)	16.55 (3.42–80.01)	<0.001	9.76 (1.61–63.19)	<0.001
Previous history of uterine anomalies	10 (17.2)	4 (1.2)	14 (3.7)	13.79 (4.47–42.49)	<0.001	13.57 (3.54–50.6)	<0.001
Previous history of psychological pressure	8 (13.8)	11 (3.4)	19 (5.0)	4.49 (1.72–11.72)	<0.001	–	–
Previous history of antiphospholipid syndrome	14 (24.1)	0 (0.0)	14 (3.7)	26.41 (15.15–100.00)	<0.001	24.59 (8.45–71.04)	<0.001

Note that *p* = probability of significance, reference category = RPL.

aOR(95%CI)^f^ and *p*-^valuef^ are for final regression models. The reference category = RPL for final model; for each risk factor, absence of the risk factor is reference.

a Logistic regression analysis adjusted for age, social status, BMI, level of education, and smoking status.

In multivariate analysis; history of unexplained RPL, endocrine disorder, uterine anomalies, and antiphospholipid syndrome were the factors independently associated with RPL.

Pregnant women who had RPL had 23.04 increased odds of having unexplained RPL as compared to pregnant women who had no RPL [AOR = 23.04; 95% CI (11.46, 36.32)]. Pregnant women who have a history of RPL had 9.76 increased odds of it being caused by endocrine disorder compared with those without prior RPL (AOR = 4.67; 95% CI: 2.33–9.37). Pregnant women who have prior history of RPL had 13.57 increased odds of it being caused by uterine anomalies as compared with those without prior RPL [AOR = 13.57; 95% CI (3.54, 50.60)]. Pregnant women who have a history of RPL had 24.59 increased probability of it being caused by antiphospholipid syndrome compared with those without prior RPL [AOR = 24.59;95% CI (8.41, 71.04)] (Table 2).

### Factors associated with recurrent pregnancy loss according to definition of RPL (the WHO/RCOG and the ASRM/ESHRE criteria)

Table 3 shows the socio-demographic characteristics of participants with prior RPL according to ASRM/ESHRE (2 RPL) and WHO (≥3 RPL) criterion. In this analysis stratified by definition of RPL (ASRM/ESHRE criterion vs. WHO/RCOG criterion) the association between RPL according to the ASRM/ESHRE vs. WHO/RCOG criterion was similar in both socio-demographic parameters without significant differences. The risk factors of RPL among participants with prior RPL according to ASRM/ESHRE (2 RPL) and WHO/RCOG (≥3 RPL) criterion is shown in Table 4. Table 5 shows the multinomial regression analysis influence of factors on RPL according to ASRM/ESHRE (2 RPL) and WHO/RCOG (≥3 RPL) criterion. In these analyses, the association between RPL according to the ASRM/ESHRE vs. WHO/RCOG criterion was similar in risk factor parameters without significant differences.

**Table 3:** Socio-demographic characteristics of participants with prior RPL according to ASRM (2 RPL) and wHO (≥3 RPL) criterion.

		2 RPL (*n* = 58)	≥3RPL (*n* = 20)	*X* ^2^	*p*
Age range (years)	<25	6(10.4)	0 (0.0)	3.77	0.15
	26–35	34 (58.6)	16 (80.0)		
	36–45	18 (31.0)	4 (20.0)		
	>45	0 (0.0)	0 (0.0)		
Marital status	Married	57 (98.3)	20 (100.0)	0.34	0.55
	Single	1 (1.7)	0 (0.0)		
Level of education	No formal education	0 (0.0)	0 (0.0)	4.33	0.11
	Primary	10 (7.2)	8 (40.0)		
	Secondary	24 (41.4)	6 (30.0)		
	Tertiary	24 (41.4)	6 (30.0)		
Social status	Upper class	12 (20.7)	8 (40.0)	5.40	0.06
	Middle class	20 (34.5)	2 (10.0)		
	Lower class	26 (44.8)	10 (50.0)		
Parity	No child	14 (24.1)	4 (20.0)	6.37	0.04
	1–4 children	36 (62.1)	8 (40.0)		
	>4 children	8 (13.8)	8 (40.0)		
BMI class	Underweight	0 (0.0)	0 (0.0)	6.94	0.03
	Normal	19 (32.8)	9 (45.0)		
	Overweight	16 (27.6)	0 (0.0)		
	Obese	23 (39.7)	11 (55.0)		

Abbreviations: BMI, body mass index.

**Table 4:** Risk factors of RPL among participants with prior RPL according to ASRM/ESHRE (2 RPL) and wHO/RCOG (≥3 RPL) criterion.

	2 miscarriages	≥3 miscarriages	OR (95%CI)	*p*-value
Unexplained	34 (58.6)	10 (50.0)	1.41 (0.51–3.93)	0.66
Advanced maternal age	18 (31.0)	4 (20.0)	1.80 (0.52–6.15)	0.93
Prior history of endocrine disorders	6 (10.3)	5 (25.0)	0.34 (0.09–1.29)	0.11
Prior history of uterine anomalies	10 (17.2)	3 (15.0)	1.18 (0.29–4.81)	0.81
Prior history of psychological pressure	8 (13.8)	0 (0.0)	8.87 (0.49–158.71)	0.19
Prior history of antiphospholipid syndrome	14 (24.1)	8 (40.0)	0.35 (0.006–18.23)	0.52

**Table 5:** Multinomial regression analysis influence of factors on RPL according to ASRM/ESHRE (2 RPL) and wHO/RCOG (≥3 RPL) criterion.

		Wald	Sig.	Exp (B)	95% CI	
					Lower Bound	Upper Bound
Age	Intercept	7.40	<0.01			
	<25			1.86 × 10^8^	1.86 × 10^8^	1.86 × 10^8^
	26–35	1.41	0.23	0.47	0.13	1.62
	36–45					
Social status	Intercept	6.59	0.01			
	Upper class	0.87	0.35	0.57	0.18	1.83
	Middle class	2.63	0.10	3.84	0.75	19.55
	Lower class					
Obesity	Intercept	4.04	0.04			
	Not obese	1.41	0.23	1.86	0.66	5.18
	Obese					

### Factors associated with recurrent pregnancy loss according to type of RPL (primary vs. secondary)

Table 6 shows the socio-demographic variables by type of miscarriage (primary vs. secondary) while the risk factors for RPL among participants with prior RPL according to type of miscarriage (primary vs. secondary) is shown in Table 7. Table 8 shows the multinominal analysis of socio-demographic variables on type of RPL. In these analyses stratified by type of RPL (primary vs. secondary), the association between primary RPL and secondary RPL was observed similarly in both socio-demographic and risk factor parameters with only advanced maternal age significantly higher in women with secondary RPL.

**Table 6:** Socio-demographic variables by type of miscarriage (primary vs secondary).

		Primary	Secondary	RPL	*X* ^2^	*p*-value
Age range (years)	<25	4 (44.4)	2 (3.40)	6 (10.4)	15.297	<0.001
	26–35	5 (55.6)	29 (50)	34 (58.6)		
	36–45	0 (0.0)	18 (31.0)	18 (31.0)		
	>45	0 (0.0)	0 (0.0)	0 (0.0)		
Marital status	Married	9 (100.0)	48 (98.0)	57 (98.3)	0.18	0.66
	Single	0 (0.0)	1 (2.0)	1 (1.7)		
Level of education	No formal education	0 (0.0)	0 (0.0)	0 (0.0)	2.37	0.30
	Primary	0 (0.0)	10 (20.4)	10 (17.2)		
	Secondary	4 (44.4)	20 (40.8)	24 (41.4)		
	Tertiary	5 (55.6)	19 (38.8)	24 (41.4)		
Social status	Upper class	0 (0.0)	12 (24.5)	12 (20.7)	3.57	0.16
	Middle class	5 (55.6)	15 (30.6)	20 (34.5)		
	Lower class	4 (44.4)	22 (44.9)	26 (44.8)		
BMI class	Underweight	0 (0.0)	0 (0.0)	0 (0.0)	1.72	0.42
	Normal	3 (33.3)	16 (32.7)	19 (32.7)		
	Overweight	1 (11.1)	15 (30.6)	16 (27.6)		
	Obese	5 (55.6)	18 (36.7)	23 (39.7)		

Abbreviations: BMI, body mass index.

**Table 7:** Risk factors for RPL among participants with prior RPL according to type of miscarriage (primary vs secondary).

	Primary (*n* = 9)	Secondary (*n* = 49)	Total RPL	OR(95%CI)	*p*-value
Advanced maternal age	3(33.3)	15(30.6)	18 (31.03)	1.13 (0.24–5.15)	0.87
Unexplained	5 (55.6)	29 (59.2)	34 (58.6)	0.86 (0.21–3.61)	0.84
Prior endocrine disorders	1 (11.1)	7 (14.3)	8 (13.8)	0.75 (0.08–6.96)	0.80
Prior uterine anomalies	3 (33.3)	11 (22.4)	14 (24.1)	1.73 (0.37–8.06)	0.49
Prior psychological pressure	4 (44.4)	15 (30.6)	19 (32.8)	1.81 (0.43–7.72)	0.42
Prior antiphospholipid syndrome	3 (33.3)	11 (22.4)	14 (24.1)	1.73 (0.37–8.06)	0.49

**Table 8:** Multinominal analysis of sociodemographic variables on type of recurrent pregnancy loss.

Parameter Estimates						
		Wald	Sig.	Exp (B)	95% CI	
					Lower bound	Upper bound
Age (years)	Intercept	1,699.94	<0.001			
	<25	433.49	<0.001	9.37 × 10^8^	1.34 × 10^8^	6.55 × 10^8^
	26–35			8.07 × 10^7^	8.07 × 10^7^	8.07 × 10^7^
	36–45					
Social status	Intercept	1,550.79	0			
	Upper class			6.23 × 10^9^	6.23 × 10^8^	6.23 × 10^8^
	Middle class	0	1	1	0	a
	Lower class					
Obesity	Intercept	6.42	0.01			
	Not obese	1.093	0.29	0.46	0.11	1.95
	Obese					

^a^ The regression coefficient vector.

### Association between type of RPL and RPL classification criterion

Table 9 shows the association between type of RPL and RPL classification criterion. The analysis shows there was no significant differences.

**Table 9:** Association between type of miscarriage and RPL classification criterion.

		2 miscarriages	≥3 miscarriages
Primary	Count	9	2
	%	81.80	18.20
Secondary	Count	49	18
	%	73.10	26.90
Total	Count	58	20
	%	74.40	25.60
		*X* ^2^	0.37
		OR (CI)	0.64 (0.12–3.23)
		*p*-value	0.59

Final regression model for factors that may predispose to types of RPL.						
		Wald	Sig.	Exp (B)	95%CI	
					Lower bound	Upper bound
Primary	Intercept	1,550.79	<0.01			
	Nulliparous		<0.01	6.23 × 10^9^	6.23 × 10^9^	6.23 × 10^9^
	1–4	0	1	1	0	—
	>4					

Reference category is secondary RPL.

## Discussion

Recurrent pregnancy loss is one of the most common infertility problems facing couples of reproductive age. Previously, there had been no study conducted in Nigeria on the prevalence and associated factors among pregnant women with a history of recurrent pregnancy loss. We have found that in pregnant women with prior history of RPL, the prevalence was 15.34% and 5.29% according to the ASRM/ESHRE and the WHO/RCOG criterion, respectively. Overall, the established significant risk factors included unexplained RPL, endocrine disorder, uterine anomalies, and antiphospholipid syndrome. A risk factor can be found in approximately 41.38% of pregnant women, while it remains unclear in the other 58.62%. This study on prevalence and risk factors of RPL is justified because the management of recurrent miscarriage should be individualized and there is currently no adequate intervention to prevent all types of recurrent miscarriage in the study environment.

The overall prevalence of RPL in this study was found to be 15.34%, which means out of hundred pregnant women around 15% of pregnant women had been affected by at least two previous pregnancy losses. This prevalence was not in keeping with the previous study conducted in Sweden with the prevalence of RPL ranges from 0.478% to 0.875%, while the mean prevalence was 0.65%. The exceedingly variability in this result could be attributed to varying age of the pregnant women at 18 years or at 42 years, respectively (7). However, when we take into account three successive losses in accordance with the WHO criterion, the prevalence was 5.29%. In a previous study, recurrent pregnancy loss affects approximately 1 to 2% of women taking into consideration three consecutive pregnancy losses occurring before 20 weeks of gestation (16). A prevalence of 0.8 to 1.4% was reported when only clinical miscarriages, i.e., pregnancy losses confirmed by ultrasound were taken into account, including biochemical losses, increasing the prevalence from 2 to 3%. The prevalence of RPL is expectedly higher in pregnant women in low and middle-income countries, because of suspected RPL or undiagnosed condition, as the required part of the diagnosis or other investigation may be lacking. Furthermore, in these low- and middle-income countries such as Nigeria, the age of fetal viability is 28 weeks, instead of 20 or 22–24 weeks agreed in high-income countries (17).

In our study, only 15.52% of the women with RPL had primary RPL while 84.48% had secondary RPL. Although comparable to Shapira et al. study in Israel that reported 39% prevalence of primary RPL and 61% prevalence of secondary RPL, the prevalence of primary RPL in our findings was lower (18). Similarly, this finding did not corroborate a previous Italian study by Vaquero et al., which had 75.70% as primary RPL and 24.30% being secondary RPL (19). In another Italian study by Ticconi et al., 65.9% of women had primary RPL while 34.1% had secondary RPL (13). Our study is also not in line with a previous study conducted in Sweden that revealed that the proportion of primary RPL and secondary RPL is 51.4% and 48.6%, respectively (6). Similarly, in an Indian study, the prevalence of primary RPL was 74.7% while secondary was 25.3% (20). The possible justification for these variations could be differences of the study population and race, diagnostic criteria, patient selection criteria, lifestyle of the participants, data collection methods and self-report nature of the study (6).

In this study, the odd of advanced maternal age is not significantly higher for pregnant women with prior RPL compared to those without RPL. This is inconsistent with report in other study in Norway (21). This finding of no significant difference for maternal age risk factor is surprising. This is because recurrent miscarriages could be due to the decreased ovarian reserve seen in advanced maternal age. Another mechanism is that advanced maternal age can lead to embryonic aneuploidy or could be due to poor egg quality leading to chromosomal (genetic) abnormalities.

Furthermore, this study showed that endocrine disorder is associated with increased chances of RPL compared to their counterparts without prior RPL. This finding is consistent with the findings of other studies and reviews (2, 3, 4, 20). This might be due to polycystic ovary syndrome, thyroid diseases, diabetes mellitus, prolactin abnormalities and implantation failure seen commonly in women with endocrine disease. Mechanisms thought to be involved are insulin resistance, hyperinsulinemia, hyperandrogenemia, or increased plasminogen activator inhibitor-1 activity (22).

In this study, pregnant women with a history of RPL had 24.59 higher odds of it being caused by antiphospholipid syndrome compared with those without a history of RPL. This finding is consistent with a previous Nigerian study by Abdulahi et al. that reported a prevalence of 14.1% for APA among women with RPL (23). Another study by Zolghadri et al. in Iran also corroborates with our findings (24). Antiphospholipid Syndrome is an autoimmune condition comprising of acquired thrombophilia and accounting for 5%–20% of recurrent pregnancy loss. The probable mechanisms of antiphospholipid antibodies causing RPL involves inducing damage of the trophoblast leading to impaired trophoblast mediated functions like spiral artery formation, secretion of growth factors, human chorionic gonadotrophin, early apoptosis of trophoblasts and abnormal inflammatory response resulting in impaired pregnancy support (25).

According to this study, the prevalence of RPL was significantly affected by the psychological pressure, but in multivariable logistic regression, adjusted for confounders, the association between RPL and the psychological pressure did not remain significant. Thus, RPL can have a significant psychological effect on the personal and professional life of the affected pregnant woman, and various feelings have been reported, such as grief and depression, hopelessness, guilt, anxiety, and anger toward the partner, friends, or the treating physician (26). Recurrent pregnancy loss has a significant psychological and emotional impact on couples (27). Several reports have looked at a possible psychological etiology for RPL, but such associations are very difficult to prove with the presence of various variables and confounding factors (7, 28).

However, some studies have showed that the causes of miscarriage that can be recognized after two pregnancy losses are like the causes that can be recognized after three consecutive pregnancy losses. For instance, a study involving 351 participants with consecutive second trimester miscarriages found the causes were idiopathic (51%), antiphospholipid syndrome (33%), cervical weakness (8%), uterine anomaly 267 (4%), bacterial vaginosis (3%), and hypothyroidism (1%) (29). Our study has confirmed similar report because the association between RPL according to the ASRM vs. WHO criterion was similarly observed in both socio-demographic and risk factor parameters without any significant differences. Therefore, it has been recommended that couples with two or more consecutive spontaneous miscarriages warrant an evaluation to identify any factor that may be associated with their poor reproductive history.

The clinical implications for these findings are that when we consider the ASRM/ESHRE criterion for RPL diagnosis, the prevalence will be significantly higher than when the WHO/RCOG definition criteria are used. However, the risk factors in each international criteria remain the same for both. This means that we can expend more resources in the use of ASRM/ESHRE than WHO/RCOG criteria, but with expectedly high chance of preventing further pregnancy loss if adequate intervention is put in place during subsequent pregnancies. The findings highlight that obstetric care providers should adopt a holistic and couple-focused approach in their prevention of subsequent RPL and include attention to the cumulative effect of multiple pregnancy losses on the woman (30). In addition, couples with RPL usually express concern about the cause and risk of recurrence. RPL requires medical intervention encompassing access to specialized clinics, investigations, and enhanced support and monitoring during future pregnancies. Most women with a history of RPL are likely to receive care from tertiary or specialist centers as they will be able to provide them with the care they need to prevent future occurrence. Women with unexplained RPL recognize that no specific cause could be identified in previous diagnostic workups after previous losses (31).

This study appears to be the first study that examined hospital based prevalence of RPL and distribution of associated risk factors in Nigeria, as no conclusive research explains the prevalence and associated factors of women with a history of RPL. These findings are vital for reproductive health policy design and program planning in low and middle-income settings. As many Nigerian states are contemplating including either two or three previous pregnancy losses in the routine antenatal high-risk program work up for RPL, our findings are important to make evidence based decisions.

Our study provided information on the novelty of the prevalence of RPL in low-income settings according to two different international and national criteria for the diagnosis of RPL. There is also the collection of comprehensive clinical data on the types and criteria for RPL. Furthermore, this research has provided baseline information on pregnant women with a history of RPL, which will help to recognize and treat underlying issues and help when allocating resources for reproductive care and preventive healthcare strategies for women with RPL.

We acknowledge certain limitations of our study. Given the cross-sectional design and our study being a single-center hospital-based study; therefore, the findings may not be applicable around the country or globally. Also, this simple cross-sectional study could not establish the determining causal relationship in all cases. Additionally, our findings should be taken with caution because the prevalence of RPL varies a lot in different studies and depends on definition of the condition (two or three losses, consecutive or non-consecutive losses, biochemical losses included or excluded) as well as the study design (cross-sectional, observational, case-control, registry and /or hospital based reports). Furthermore, the lack of comparison groups limits its control over unobserved heterogeneity among respondents. Furthermore, although some women were referred with prior diagnosis of RPL, we did not have access to data on diagnostic measures used to confirm various risk factors or causes. Most of the miscarriages were self-reported and it is based on questionnaires completed by the participants, with the known methodological problems of potential recall and selection bias. Even though 30% of pregnant women initially approached for screening did not participate in the study, we were able to recruit a relatively large sample of participants. Lifestyle and obesity also play an important significant role in RPL, although this was not revealed in our present study (32). Another limitations of the study was that we did not obtain any information regarding the paternal factors as well as information regarding cases reported with problems in the male counterpart with regards to smoking, obesity and diabetes mellitus in the absence of male factor abnormalities. This is because recent studies have shown that advanced paternal age is also associated with an increased risk of spontaneous recurrent miscarriage (33–35).

## Conclusion

The prevalence of RPL was 15.34% and 5.29% according to the ASRM/ESHRE and the WHO/RCOG criterion respectively, with secondary type predominating. Unexplained loss, endocrine disorder, uterine anomalies and antiphospholipid syndrome were significantly associated with RPL. No significant differences with regard to risk factors were seen according to the two different international diagnostic criteria except advanced maternal age which was significantly higher in secondary type of RPL than in the primary type. Further research is needed to confirm our findings and to better characterize the magnitude of the differences in the prevalence and any possible differences in the risk factors according to different national and international criteria for RPL.

## Data Availability

The original contributions presented in the study are included in the article/Supplementary Material, further inquiries can be directed to the corresponding author.
